# “It’s the worst thing I’ve ever been put through in my life”: the trauma experienced by essential family caregivers of loved ones in long-term care during the COVID-19 pandemic in Canada

**DOI:** 10.1080/17482631.2022.2075532

**Published:** 2022-05-30

**Authors:** Charlene H. Chu, Amanda V. Yee, Vivian Stamatopoulos

**Affiliations:** a Lawrence S. Bloomberg Faculty of Nursing, University of Toronto, Toronto, Ontario, Canada; b KITE Research Institute, Toronto Rehabilitation Institute, University Health Network, Toronto, Ontario, Canada; c Faculty of Arts and Science, University of Toronto, Toronto, Ontario, Canada; d Faculty of Social Science and Humanities, Ontario Tech University, Oshawa, Ontario, Canada

**Keywords:** COVID-19, essential family caregivers, focus groups, long-term care home, nursing homes, trauma

## Abstract

**Background:**

Essential family caregivers (EFCs) of relatives living in long-term care homes (LTCHs) experienced restricted access to their relatives due to COVID-19 visitation policies. Residents’ experiences of separation have been widely documented; yet, few have focused on EFCs’ traumatic experiences during the pandemic. Objective: This study aims to explore the EFCs’ trauma of being locked out of LTCHs and unable to visit their loved ones in-person during COVID-19.

**Methods:**

Seven online focus groups with a total of 30 EFCs from Ontario and British Columbia, Canada were conducted as part of a larger mixed-method study. We used an inductive approach to thematic analysis to understand the lived experiences of trauma.

**Results:**

Four trauma-related themes emerged: 1) trauma from prolonged separation from loved ones; 2) trauma from uncompassionate interactions with the LTCH’s staff and administrators; 3) trauma from the inability to provide care to loved ones, and 4) trauma from experiencing prolonged powerlessness and helplessness.

**Discussion:**

The EFCs experienced a collective trauma that deeply impacted their relationships with their relatives as well as their perception of the LTC system. Experiences endured by EFCs highlighted policy and practice changes, including the need for trauma-centred approaches to repair relational damage and post-pandemic decision-making that collaborates with EFCs.

## Introduction

Early on in the COVID-19 pandemic, long-term care homes (LTCHs) and other congregate-care housing facilities implemented restrictive visitation policies (also known as “lockdown policies”) that prevented visitors, including essential family caregivers (EFCs), from entering the facilities (Chu et al., 2022; Government of British Columbia, 2022). The Organization for Economic Co-operation and Development’s (OECD) policy brief report that assessed the LTC policies during the pandemic in all the OECD countries reported that similar restrictions to visitors was common, with 32 OECD countries (e.g., U.K., U.S., Japan) implementing such policies, and 17 of these countries also mandating that all meals be eaten in bedrooms (OECD, 2021). The LTC sector was “ill-prepared to tackle a health emergency” (OECD, 2021, p. 2) since acute care was prioritized in the initial phases of the pandemic to receive access to testing and personal protective equipment (PPE) (OECD, 2021). Scholars in ethics and caregiver advocates have critiqued the visitation policies, referring to them as reactionary policies to a system-level “failure to plan” in preparing LTCHs (Faghanipour et al., 2020, p. 1171) to protect residents and staff from COVID-19 infection. The situation in LTC homes was so dire in at least four countries (e.g. Canada, the Czech Republic, Estonia, Germany), that the army was deployed to the LTCHs to provide assistance with daily care (OECD, 2021).

During the pandemic, Canada implemented multiple policies to create an “iron ring” or “island” around LTC homes that involved restricting all non-essential visitors (National Institute on Ageing, 2020). In the first and second waves of the pandemic, it was unclear when these strict policies would be relaxed which caused significant social isolation and emotional harm to residents (Chu et al., 2020; Chu, Wang et al., 2021). Yet, despite these policies, Canada had the highest proportion of LTC deaths out of all the OECD countries with over 14,000 deaths occurring in LTCHs by February 2021 representing more than two-thirds of Canada’s overall COVID-19 deaths (Canadian Institute for Health Information [CIHI], 2021). The percentage of COVID-19 deaths in LTC was consistently double the OECD average throughout the COVID-19 pandemic (Morciano et al., 2021).

In Ontario, Canada’s most populous province with 14.5 million people, LTCHs were particularly hard hit (CIHI, 2021) such that the army in addition to healthcare providers from local hospitals were deployed to multiple LTCHs with outbreaks. During this time when there was a staffing crisis, families were unable to access their relatives to provide care. Some LTCHs initially offered virtual visits as a means for families to connect with residents (e.g., April 2020). Ongoing public scrutiny of the separation surrounding visitor access resulted in outdoor visitations (e.g., resident and family meeting outdoors) from June to August 2020 before family members were eventually granted indoor access in September 2020. Each resident was allowed to designate up to two “essential caregivers”, as long as they completed infection prevention and control (IPAC) training and wore full PPE (e.g., mask, gloves, gown; Ministry of Long-Term Care [MOLTC], 2020). Another province with high case rates was British Columbia (BC). Although rates of severe acute COVID-19 infection and mortality among LTC residents were several times higher in Ontario than BC in the first wave of the pandemic (Liu et al., 2020), lockdown policies remained in the majority of LTCHs for one year (March 2020 to April 2021). In some jurisdictions, EFCs were recognized only when their loved ones were in palliative care (Government of British Columbia, 2020; Williams, 2020). It was not until March 2021 that BC provincial policy was amended to also allow for up to two visitors (without requiring supervision) and the addition of physical touch between resident and visitors, with IPAC measures in place. Importantly, and similarly to Ontario, these expanded social visits only applied when the home was not in an outbreak and required advanced booking, visitor health screening, and PPE use.

The widespread involvement of family in LTCH is well documented (Garity, 2006; Gaugler, 2005; Gaugler et al., 2000), as family caregivers are often involved in hands-on care tasks (e.g., dressing, toileting, meal assistance), monitoring changes to loved one’s health status, advocating on behalf of the resident, and providing oversight to their loved ones personal and instrumental care (Baumbusch & Phinney, 2014; Gladstone et al., 2006; Port et al., 2005; Powell et al., 2018; Zimmerman et al., 2013). Moreover, family involvement in LTCHs has been shown to reduce staff burden, resident mortality, infection, and hospitalization (Gaugler, 2005). Despite family caregivers providing essential daily care, and emotional and psychological support (National Academies of Sciences, Engineering and Medicine, 2016; Schlaudecker, 2020; Stall et al., 2019), their critical role was not established as distinct from “general visitors” in these policy being implemented in LTCHs (Drury, 2020; Stall et al., 2020). To counter this lack of recognition, the “essential caregiver” terminology was coined in Ontario as a deliberate opposition to the categorization of families as “nonessential visitors”. A grassroots online #MoreThanAVisitor campaign (CTV Ottawa, 2020) was initiated to advocate for more EFC access (Drury, 2020; Stall et al., 2020). In the political sphere, the creation of Bill 203 (“More Than A Visitor Act”) tabled by Lisa Gretzky, a Member of Provincial Parliament, was seeking the legislated recognition and reinstatement of EFCs into LTCHs and other congregate care living settings across Ontario (Gretzky, 2020). Ultimately, the visitation restrictions removed an incalculable source of informal care and support provided by informal family caregivers within a chronically underfunded and fractured Canadian LTC sector, a fact reiterated by the commissioners of Ontario’s Long Term Care COVID-19 Commission (Marrocco et al., 2021). Despite the often highly involved role family members play in LTCHs, family caregivers have often been portrayed as “visitors” and positioned as “outsiders” in an institutional context that reinforces power inequities between family and LTCH staff/administration who are deemed “insiders” (Baumbusch & Phinney, 2014).

The Ontario Patient Ombudsman (2020) report referred to COVID-19 as a “crisis in LTC” (p. 6), with many distressing situations documented about family caregivers being denied access to their loved ones in LTCH and relatives dying alone. Media outlets consistently documented the widespread detrimental effects of lockdown policies on LTCH families across Canada and the accounts of disturbing neglect and preventable death occurring in the families absence (Harris, 2020; Howlett, 2021; Kirkup, 2020; Mahoney, 2020; Mauro, 2020; Roumeliotis & Mancini, 2020). Advocates and researchers suggested that the policies keeping EFCs out of LTCHs created a crisis of social isolation in LTCHs (Abbasi, 2020; The New York Times, 2020) one that focused primarily on quantity versus quality of life (Chu et al., 2020) as residents were confined to their rooms with minimal to no social contact for months. There is a large body of evidence that previously established that social isolation increases older adults’ risk for anxiety, depression, cognitive impairment, and mortality (Holt-Lunstad et al., 2015; Novotney, 2019). For older adults with chronic, disabling, or serious health conditions, family and other unpaid caregivers are the most important source of emotional and practical support (Hado & Feinberg, 2020; National Academies of Sciences, Engineering and Medicine, 2016, 2020; Reinhard et al., 2019). And while the trauma suffered by residents from the COVID-19 lockdown policies and the resulting social isolation has been previously described in the literature (Abbasi, 2020; Chu et al., 2020; Chu, Wang et al., 2021; Diamantis et al., 2020; Faghanipour et al., 2020; Simard & Volicer, 2020; Wu, 2020), little research has focused on the traumatic experiences incurred by EFCs of persons residing in LTCHs during COVID-19.

The COVID-19 pandemic has been viewed as the cause of collective trauma described as “a cataclysmic event” and “crisis of meaning” experienced by a cohort (Hirschberger, 2018, p. 1441) that results in individual and group level negative psychological consequences (Griffin, 2020; Lund et al., 2020; Masiero et al., 2020; Silver, 2020; Stanley et al., 2021; Taggart et al., 2021). Trauma is characterized by “complex emotional responses to a stressful event, that overwhelms one’s capacity to cope” (Masiero et al., 2020, p. 514). Psychological responses to trauma are influenced by several factors including circumstances and resources (Silver, 2020), and disproportionately impact marginalized communities. Family caregivers, who are predominately women, are vulnerable to trauma from the act of providing care, being empathetic, and exposure to “stress resulting from helping or wanting to help” (Figley, 1995, p. 10). Additionally, negative health consequences such as social exclusion, social isolation, financial disruptions, and emotional distress is associated with caring for relatives with cognitive disorders (Ae-Ngibise et al., 2015) increasing their vulnerability to the negative emotions related to trauma. While the experiences and responses to COVID-19 are heterogeneous, similarities can be observed in the psychosocial consequences experienced by individuals and groups (Masiero et al., 2020). In order to address trauma, the experiences of trauma must be acknowledged because minimizing the long-term damage contributes to the maintenance of the policies and structures that caused the trauma (Hübl & Avritt, 2020). Documenting the collective traumatic experiences of EFCs during COVID-19 will inform the public discourse surrounding pandemic mandates and has contextual and situated implications on how to rebuild the LTC sector post-COVID-19. The purpose of this study is to explore the trauma experienced by EFCs with loved ones in LTCHs during COVID-19, in doing so shedding light on the unique challenges experienced by a group whose meaningful access to loved ones was curtailed during COVID-19.

## Methods

As part of a larger mixed-methods study, this qualitative paper examines the trauma experienced by EFCs during COVID-19 visitor restrictions. The ongoing larger mixed-methods study aims to understand the lived experiences of both caregivers and residents during the COVID-19 visitor lockdown policies to gain insights about the consequences of these policies.

The paper followed the Consolidated Criteria for Reporting Qualitative Research (COREQ) checklist (Tong et al., 2007). Ethics approval was sought and approved by the Ontario Tech University’s (REB #16086) and the University of Toronto’s (REB #40070) Research Ethics Boards. Informed consent was collected electronically by the PIs prior to the focus groups. All digitally recorded focus groups (FGs) were kept in a secured research server at the universities. Participants were provided a $100 digital gift card to their choice of a Canadian bookstore or an online marketplace for volunteering approximately 2-hours of their time to fill out an online survey in addition to completing the 90-minute focus group interview. This was deemed an adequate honorarium by the University Research Ethics Boards that would not produce an undue influence to participation for the adults included in the sample.

It was anticipated that participants would have strong emotions when sharing their experiences in the focus group. The PIs reminded participants prior to initiating the FG that a list of resources about trauma support and distress was available, and that they could feel free to contact us at any time to request the resources. We also made it clear that participants could take a break at any time during the FG by muting, turning off their camera, or exiting the Zoom platform.

### Participant recruitment

Caregivers are defined as, “a type of essential visitor who is visiting the home to provide direct care to meet the essential needs of a particular resident […] [they] must be designated by the resident or his/her substitute decision-maker” (Government of Ontario, 2021a). This paper will focus on EFCs, referring to the resident’s family member. Recruitment took place only on Twitter, whereby digital recruitment posters were shared on the principal investigators’ (PIs) accounts which were then retweeted by large professional organizations and the universities where the PIs work. The eligibility criteria were as follows: Canadians that self-identified as EFCs whose LTCH access was affected by the pandemic visitation policies, able to communicate in English, and have a stable internet connection. Twitter provides an instantaneous media platform that is cost-effective to use and reaches a wide audience (O’Connor et al., 2014). Eligible participants connected with the PIs via email, and further instructions about the study along with a consent form indicating the participant’s legal rights were shared via email. PIs offered telephone calls or video chats if interested individuals wanted to ask additional questions to ensure informed consent. Purposive sampling was used when recruiting participants, with an attempt to enrol not only EFCs of residents in Canadian LTCHs but also an equal number of males and females. The PIs used additional social media posts encouraging male caregivers to participate, and extended the recruitment period by two weeks in hopes that the targeted tweets and additional time would assist in recruiting more males. Despite these efforts, the vast majority of interested individuals were females. Also, while recruitment was open to all EFCs in Canada who could speak English, interested participants were from Ontario and BC. This may reflect the fact that these provinces had the highest death rates out of the English-speaking provinces (CIHI, 2021) and/or retained some of the more restrictive lockdown policies across Canada, as was the case in BC.

### Data collection

Thirty EFCs from Ontario and BC were recruited and participated in an online survey on Qualtrics answering questions about their sociodemographic information (e.g., gender, age, employment status, relationship to their loved one in LTCH), details about EFCs loved ones’ LTCH (e.g., duration living in LTCH, LTCH’s profit status, room type), their visitation experiences pre- and post-pandemic, and the types of unpaid care they provide to their loved ones. Finally, they how often they experienced stress-related symptomology related to visitor restrictions to LTCH, coping methods, and the types of support they sought out to manage these experiences. The online survey was completed prior to the FGs.

Focus groups were the primary qualitative method of data collection. Seven 90-minute online FGs (N = 30) via Zoom were held with four to five EFCs per group. The number of people selected in each FG is within the ideal size (Krueger & Casey, 2014), and were formed according to the joint availability and schedules of participants and researchers. The FGs were conducted between January to March 2021. Focus groups are an optimal approach for conducting exploratory research in an area with an established scarcity of scholarship. The interactions within group discussions enable participants to share a collective experience and build off each other’s stories (Sagoe, 2012), in doing so FGs can empower participants (Van den Hoonaard & Van den Scott, 2015). Empowering participants was deemed particularly important in this context given the collective disempowerment of caregivers due to prolonged visitor restrictions during COVID-19. The virtual aspect of the focus groups was necessitated by public health measures to limit in-person group meetings, but provided some benefits. For example, the virtual nature of the groups afforded some scheduling benefits to participants who were working, providing child care, and coordinating their visits to the LTCHs. Participating in in-person research would not have been feasible for many of the participants as these caregivers were required to complete weekly negative COVID-19 tests via nasopharyngeal swabs and were extremely diligent to stay away from any potential COVID-19 risks. Further, it provided participants the options to minimize their audio and/or video if they needed a break from discussing difficult matters in the FG.

One PI (VS) led the FG discussion as moderator given her recognized and trusted public advocate role for LTC caregivers throughout COVID-19 and extensive background in conducting qualitative research. The other PI (CC) also has a background and training in qualitative methodology and was responsible for taking field notes and asking clarifying questions. Given the extensive advocacy work of both researchers leading up to this study which involved speaking to hundreds of family caregivers across the country, the primary interest in the research topic was to illuminate the subjective meanings and LTC context in order to explore the extent of the collective harm and trauma experienced by EFCs of residents living in LTCHs. Both PIs have their PhDs and are female professors at Ontario-based universities. As part of our reflexive practice, we kept notes where we acknowledged our personal and social standpoints, and positioning throughout all phases of the study.

The PIs used a semi-structured, pilot tested FG guide with open-ended questions. Using an interpretivist lens, FGs allowed participants to explain their lived experiences to share their stories in their own words using their own terms (Goldkuhl, 2012). Interview questions included “describe your experience as a caregiver for your loved one in LTC during the pandemic?” and “describe what it was like to see your loved one for the first time” in order to understand their subjective meanings of trauma experienced by EFCs. According to Goldkuhl (2012) this means researchers illuminate participants’ understandings and social contexts, as understood by them and avoid distorting their words and terms.

To ensure confidentiality, the PIs required participants to select a pseudonym or a randomly assigned a number prior to the start of the focus group. The PIs renamed the participants in Zoom and instructed the participants to use their pseudonyms if they were to refer to each other. We informed them that we would using them throughout the FG video and audio recording on Zoom. All participants were comfortable with using and being referred to as their pseudonym or number without any concerns, and understood the importance of confidentiality. All participants had their cameras on throughout the duration of FGs and understood that they were being recorded to ensure that body language and emotions would be accurately transcribed. All names and LTCHs were removed to anonymize the transcripts. After each FG, the PIs went over the key themes and points brought up during the FGs. The PIs discontinued conducting further FGs only when no novel concepts were generated during the discussions (i.e., saturation; Glaser & Strauss, 1967). In this study, saturation was achieved after seven FGs. Neither PIs had any personal relationships with the 30 participants prior to the study. There was no attrition and no repetition of FGs over the course of the study.

### Data analysis

**Figure 1.**
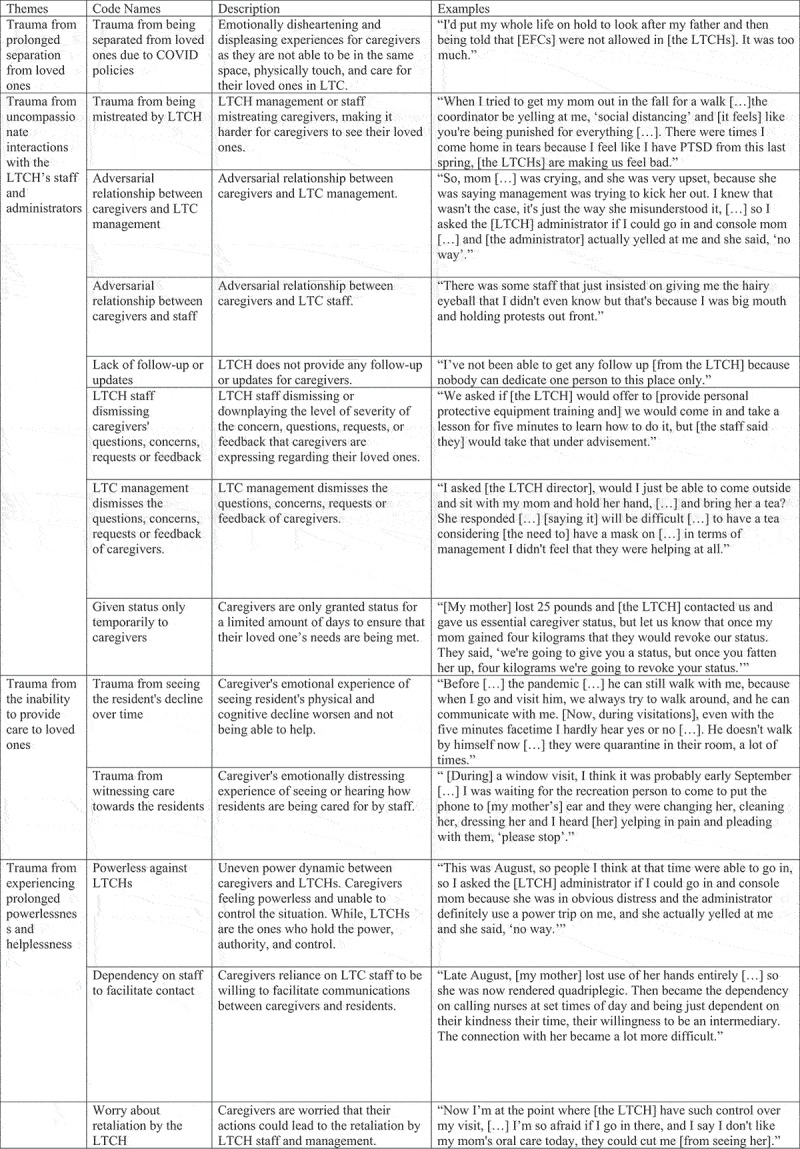
Coding tree.

Given the exploratory nature of this study, an inductive approach to analysis was utilized. In line with Thomas (2006), inductive analysis refers to “approaches that primarily use detailed readings of raw data to derive concepts and themes”. As such, our analysis was driven by the experiences of our participants. One project researcher (AY) was tasked with transcribing and in the event that the pseudonyms or number were not used, the transcripts were anonymized, by removing the participants’ actual name. The NVivo 12 software was used to store and code the transcripts. Braun & Clarke’s work (Braun & Clarke, 2006) informed our line-by-line thematic analysis of the data. Braun & Clarke formulated a six-step process that includes getting familiar with the data, formulating the codes, finding the themes based on recurring patterns, assessing the themes, describing the themes, and drafting the research paper (Braun & Clarke, 2006). All the researchers were familiar with the data either through facilitating or transcribing the FGs verbatim. We generated the initial set of codes based on field notes and observational notes. These initial codes were generated independently between researchers and compared to ensure trustworthiness and credibility (Anney, 2014). Then similar codes or patterns across the data were organized into themes, and similar themes were collapsed. The researchers generated a coding dictionary and coding tree which documented the name of the themes along with their descriptions. Researchers held bi-weekly meetings to refine the themes, by reading the quotes and discussing the themes. Figure 1 illustrates the coding tree. For the sociodemographic information and caregiver attributes, descriptive statistics were calculated and these participant characteristics are reported in the following section. The results of the online surveys did not inform the analysis of the qualitative data nonetheless the items of the survey pertaining to stress symptomology (e.g., feeling sad, anxious, tired) were complementary to the interview questions (e.g., “how has your experience of having a loved one in LTC affected you emotionally and/or physically?”)

### Participant characteristics

The participants’ characteristics and details about the LTCH facility are summarized in Table I. Of the 30 EFCs, there were 29 females and one male, despite efforts to recruit more males. The uneven gender representation in our sample reflects how caregivers are often women. According to Statistics Canada (2018), 54% of women are caregivers compared to 46% male caregivers, the proportion of women who are unpaid family caregivers is even higher within LTCHs (Estabrooks & Keefe, 2020). Twenty-one of the 30 EFCs were from Ontario, and the remaining 9 EFCs were from BC. Participants were mostly employed (63%), caring for their parents in LTCHs (80%), and predominantly in the 55–64 age range (50%). Loved ones most commonly resided in publicly owned (municipal) LTCHs (50%), while the proportion of those living in private for-profit and private non-for-profit LTCHs was 27% and 10%, respectively. The majority of the residents resided in their LTCHs for one to two years (48%) or three to five years (37%), with 80% of them living in private rooms.

**Table I.** Descriptive characteristics of study participants and LTCH facility (N = 30)

Characteristic	N (%)
Province of Residence	
Ontario	21 (70)
British Columbia	9 (30)
Gender	
Female	29 (96.7)
Male	1 (3.3)
Age	
35–44	3 (10.0)
45–54	8 (26.7)
55–64	15 (50.0)
65+	4 (13.3)
Employed	
Yes	19 (63.3)
No	11 (36.7)
Relationship to LTC resident	
Daughter	23 (76.7)
Son	1 (3.3)
Spouse	5 (16.7)
Grandchild	1 (3.3)
Resident’s length of stay	
<1 year	2 (6.7)
1–2 years	14 (46.7)
3–5 years	11 (36.7)
5+ years	3 (10.0)
Profit status of LTC home	
Private, not-for-profit	3 (10.0)
Private, for-profit	8 (26.7)
Publicly owned (municipal)	15 (50.0)
Unsure	4 (13.3)
Room type	
Private room	24 (80)
Semi-private room	6 (20)

Table II outlines attributes of EFCs’ pre- and post-pandemic visits including satisfaction with care provided by the LTC home as well as the care tasks they would perform. Before COVID-19, the majority of EFCs would visit their loved ones in the LTCH more than four times per week (56.6%). The average duration of a visit was reduced from 135 minutes before COVID-19 to 81 minutes over the course of the pandemic. The majority of EFCs “agreed” (30%) or “neither agreed nor disagreed” (30%) to the following statement, “I was happy with the care my loved one was provided by the nursing home *before* the pandemic”. In a similar statement, “I was happy with the care my loved one was provided by the nursing home *during* the pandemic”, the most common response among EFCs was “disagree” (33%) and “strongly disagree” (30%). EFCs reported providing a wide range of support to their family members with the most common responsibilities being “supporting personal hygiene” (87%), “supporting cognitive stimulation” (97%), “supporting meaningful connection and/or relational continuity” (97%), and “supporting leisure activities that the resident enjoys” (83%). Importantly, the vast majority of participants (87%) have not been able to resume the full range of care tasks they provided pre-pandemic.

**Table II.** Survey responses from family caregivers about visits to the nursing homes before and during the COVID-19 pandemic (N = 30)

Question/Statement	N (%)
On average, how many times a week did you visit your loved one in LTC *before* COVID-19?	
0–3 times per week	13 (43.3)
4–7 times per week	10 (33.3)
7+ per week	7 (23.3)
What was the average duration of your visits to the LTC home (in minutes) *before* COVID-19?(Mean, St. deviation, Range)	135 (72.4,0–360)
What was the average duration of your visits to the LTC home (in minutes) *during* COVID-19? (Mean, St. deviation, Range)	81 (75.6, 0–300)
I was happy with the care my loved one was provided by the nursing home *before* the pandemic	
Strongly agree	4 (13.3)
Agree	9 (30.0)
Neither agree nor disagree	9 (30.0)
Strongly disagree	4 (13.3)
Disagree	4 (13.3)
I was happy with the care my loved one was provided by the nursing home *during* the pandemic.	
Strongly agree	1 (3.3)
Agree	5 (16.7)
Neither agree nor disagree	5 (16.7)
Strongly disagree	9 (30.0)
Disagree	10 (33.3)
What kind of unpaid care and support would you provide to your loved one when visiting pre-pandemic? Check all that apply.	
Supporting cognitive stimulation (e.g., talking about family events, discussing memories etc.,)	29 (96.6)
Supporting meaningful connection and/or relational continuity (e.g., providing company etc.,)	29 (96.6)
Supporting personal hygiene (e.g., grooming, nail care, oral care)	26 (86.6)
Supporting leisure activities that the resident enjoys (e.g., playing games together, hobbies)	25 (83.3)
Supporting resident’s communication with others in the home (e.g., other residents or staff)	24 (80)
Cleaning/housekeeping (e.g., of the residents’ room etc.,)	24 (80)
Supporting mobility (e.g., helping them to walk, get out of bed)	21 (70)
Supporting feeding	15 (50)
Supporting toileting (e.g., changing incontinence products, helping resident to the bathroom)	14 (46.6)
Preventing adverse events (e.g., turning or repositioning to prevent pressure ulcers, person-centred care to prevent responsive behaviours, supervision to prevent falls)	13 (43.3)
As of February 2021, have you been able to resume the full range of support tasks that you usually provided during COVID-19?	
No	26 (87)
Yes	4 (13)

Table III reveals the EFCs daily stress-related changes to their body, actions, or thinking, as a result of their LTCH experiences during the pandemic. The most common stress-related changes included daily muscle tension (57%), feeling powerless (53%), feeling sad (50%), feeling tired (47%), feeling worried, confused or anxious (40%) and feeling physically exhausted (37%). As a result of the stress-related changes 70% of EFCs sought assistance from family or friends (49%), as well as mental health and/or other medical professionals (e.g., family doctors) (44%).

**Table III.** Stress related symptomology among essential family caregivers resulting from LTC experiences during COVID-19 pandemic (N = 30)

Question/Statement	N (%)
People experiencing prolonged stress can undergo noticeable changes in their body, actions and/or thinking. Please indicate how often you have felt the following as a result of your LTC experiences during Covid-19. (Check all that apply)^a^	*Daily incidence*
My muscles feel tense	17 (56.7)
I feel powerless	16 (53.3)
I feel sad	15 (50)
I feel tired	14 (46.7)
I feel tired	14 (46.7)
I feel worried and confused	12 (40)
I feel anxious	12 (40)
I feel physically exhausted	11 (36.7)
I feel guilt or like a failure	9 (30)
I am having headaches or stomach aches	8 (26.7)
I am sleeping less	8 (26.7)
I am having trouble concentrating, remembering or making decisions	8 (26.7)
I am angry and irritable	8 (26.7)
I am drinking more coffee	6 (20)
I keep fidgeting	6 (20)
I am eating more	5 (16.7)
I feel like I can’t cope	5 (16.7)
I don’t want to do any work	5 (16.7)
I am eating less	4 (13.3)
I feel sick or in physical pain	4 (13.3)
I am using alcohol, cigarettes, or other drugs to help me cope	4 (13.3)
I have had diarrhoea	4 (13.3)
I lose patience with people	4 (13.3)
I feel strains in my relationships at work	2 (6.7)
I feel strains in my relationships outside of work	2 (6.7)
I have bad dreams	2 (6.7)
Have you sought assistance for any of these issues noted above?	
Yes	21 (70)
No	9 (30)
Who have you turned to for emotional and psychological support during this time? (Check all that apply)^a^	
Family or friends	20 (48.7)
Physician (e.g., family doctor, walk-in clinic, emergency)	10 (24.3)
Mental health professional	8 (19.5)
Support groups (e.g., advocacy groups, spiritual activities)	2 (4.8)
Other (e.g., pet therapy)	1 (2.4)

^a^The percentages are calculated out of 41 responses as participants were able to select multiple answers to the question.

## Results

Trauma was consistently identified and described in each FG by the participants when asked to describe their experience during the lockdown policies. From the data, four unique but interrelated themes connected to trauma during EFCs visitations experience were identified: 1) Trauma from prolonged separation from loved ones; 2) Trauma from uncompassionate interactions with the LTCH’s staff and administrators; 3) Trauma from the inability to provide care to loved ones; and, 4) Trauma from experiencing prolonged powerlessness and helplessness.

### Theme 1: trauma from prolonged separation from loved ones

Restricted access to residents in LTCHs over the course of the pandemic was repeatedly noted by participants as one of the hardest aspects to emotionally endure during COVID-19. EFCs’ inability to see and communicate with their loved ones living in LTCHs caused a significant amount of anxiety and fear. Participants described “lost time” and the persistent presence of long-term negative emotions. As demonstrated by these three EFCs:

Not seeing your loved one who you saw on a daily basis, and to hear the decline, and not be able to set eyes on them, it will just be with you forever. (EFC ‘1’, FG3)

Imagine being locked away from your husband. Never to see your husband. Locked away from your parent is horrible, but I was locked away from my husband! This is my other half - this is half of my life, and they’ve stolen it (EFC ‘4’, FG6)

We’ve lost an entire year with our loved ones and you can’t get that back and that’s the disheartening thing […] Having lost a year, with no hope on the horizon, I feel like we’ve just lost the last bit of time with her. I just want to hug her. (EFC ‘2’, FG4)

Consequently, the ongoing separation damaged some relationships between the EFCs and their loved ones. EFCs revealed that their loved ones with dementia would become visibly upset from the visitations due to the frequent changes to the types of visits (e.g., online, window, outdoors), and the frequency and duration of visits. EFCs also reported that residents would often become confused and frustrated during visits because they did not understand why EFCs were unable to come inside the LTCH or within arms reach. EFCs reported that witnessing and causing such distress to their loved ones “tore them apart”. For example, one participant describes her experience and feeling emotionally broken by the end of one particular visit:

There was one time at a window visit, one of the more painful [visits], towards the end where [my mother] was pleading with me to come beside her, to be beside her bed, and to not leave her. She would say to me, ‘you’re the only person I feel safe with’ […] It just broke me. (EFC ‘Morgan’, FG1)

The provision of virtual and/or outdoor visits in the first three months of the pandemic did little to reconnect EFCs and loved ones. Participants recalled visitations that were ineffective as LTCHs did not consider the resident’s impairments and stringent COVID-19 protocols created an emotionally unpleasant visitation. One EFC described the impacts of such visitations on her mother:

[My mother] got really advanced dementia, to the point where she’s practically comatose and really the only connection I have with her is to hold her hand or stroke her arm. In the lobby [of the LTCH] we’re not permitted to do that. We had to stay on opposite sides of the plexiglass and it was very distressing for her. (EFC ‘5’, FG4)

During the various iterations of visitations, participants described feeling emotionally traumatized as some residents exhibited an overwhelming sense of “despair”, “sadness” and “distress” by the end of a visit, given that visits were often only up to 30 minutes, once a week. EFCs would recount instances of crying in their cars after visits. Other EFCs stopped visiting to avoid the emotional distress that can be inflicted on their loved ones and themselves:

I was doing the window visits, but I had to stop because, when [my husband] saw me, he went absolutely frantic. [He] couldn’t understand why I wouldn’t go in and was trying to rip the window frame out. He’s a strong man. I had to go, it was so traumatic because he’s on the ground floor and [he’d] run up and down the corridor and go into other people’s rooms and wrestle the window frames there to keep [his eyes on me]. (EFC ‘5’, FG5)

### Theme 2: trauma from uncompassionate interactions with the LTCH’s staff and administrators

EFCs perceived their interactions with the LTCHs’ staff and administrators as uncompassionate and unempathetic when turning to them for support and answers. Numerous participants reported feeling “disrespected”, “ignored” or that staff wanted to “keep them out” for fear of conditions in the homes being reported the media or other authorities. Interactions with LTCH staff made EFCs feel othered as if they were the “new enemy”. Whether it be from frontline staff or owner/operators, there was a clear sentiment of “us versus them” described by participants which was a glaring shift from feeling like EFCs and staff were on the same team:

Where did that schism happen? At what point did we become an overnight enemy to the staff […] It just boggles my mind that in a heartbeat [we] became something to be scared of, the enemy, something to be feared. They wanted to keep us out. (EFC ‘1’, FG3)

Uncompassionate interactions experienced by EFCs were in the form of fractured, incomplete and oftentimes, dismissive communication from staff. Participants would discuss repeated attempts to contact the homes for updates only to be met with busy signals or voicemails. Below, two EFCs recounted the lack of communication and inconsistent communication with LTCH personnel:

It’s the lack of communication. When [my mother] had COVID, maybe we got a phone call maybe we didn’t. If we didn’t get one did that mean she was okay or not okay, wondering if they forgot, there was no weekly news bulletin, nothing came up and there was no open form of communication. It was never laid out if you had an issue, you contacted this person […]. They kept switching roles, so the activity director was then doing the Zoom calls, but then you’d get contacted by somebody else. (EFC ‘2’, FG4)

My mom started to really [deteriorate] … [I tried calling] the [LTCH’s] clinical director, one more time. She picks up. It was very tasty. She skirts [my questions] and before we go any further [I asked the clinical director], ‘why have I not heard back from you for six weeks or from the associate director?’. [The clinical director] totally glossed over [my questions] and says basically” we’re busy and your opinion is derriere.’ (EFC ‘Morgan’, EFC 1)

In addition to the lack of communication and transparency, participants recalled how they felt like they weren’t heard or being listened to. For instance, when they tried to be helpful, their suggestions to improve visitations were ignored:

[After] six months, I got my first visit with her in the lobby. [But the staff] forgot to put her hearing aids in, even though I warned them everyday for about five days leading up [to the visit]. I was so excited … [but] she couldn’t hear me and she’s very distracted. And so I started making suggestions about how [the home] could maybe switch this with microphones, walkie talkies [to improve residents’ ability to communicate] […] they could put us in a room where the delivery guys don’t come through with their carts [in the lobby]. (EFC ‘2’, FG4)

Prior to visitations, EFCs reported that staff from LTCHs did not notify them about the home’s outbreak status resulting in last-minute cancellations of visits. Consequently, many EFCs felt frustrated and disappointed in not being able to see their loved ones. One participant described how she was not informed that a facility outbreak was over which resulted in missed opportunities to spend time with her critically ill mother who subsequently passed away:

I told [the LTCH clinical director] ‘I’ve been trying to reach you to ask for permission to be at the bedside with my mother’ and she says to me, ‘oh, we were cleared from the outbreak two weeks ago. You know, you could have been in two weeks ago.’ They never told me … Honestly, I think I will never forgive them for that. I feel like it was punitive because I’m such a big mouth and I’ve called the [LTCH’s] hotline so many times. I’ll never forgive them for that. I had four visits with [my mom] before she died. It was cruel. (EFC ‘Morgan, EFC1)

EFCs reported that the infrequent updates of visitation policies or cancellations by LTCHs were incompatible with EFCs’ obligations and duties, such as full-time work or caring for young children or a sick spouse. For instance, EFCs expressed their inability to schedule visitations outside of working hours which was highly problematic as the majority of the participants were full-time workers:

I did get a call Monday of last week that [the LTCH] were allowing essential caregivers to come in again, but you had to schedule a visit and they only had certain dates and times available […] it’s hard when you work (EFC ‘4’, FG3)

When EFCs would complain to the staff about their scheduling conflicts, they were not met with compassion. Instead, EFCs felt like they needed to make the difficult decision to either miss work or see their loved ones. This constant battle to accommodate their schedules to meet the availabilities of the homes was mentally and emotionally draining for EFCs in this study.

### Theme 3: trauma from the inability to provide care to loved ones

The EFCs described the pain of seeing their loved ones deteriorate all while knowing they could have helped if they were allowed in. Many EFCs were not merely the powers of attorney and/or substitute decision-makers for LTCH residents but more importantly, they self-identified as the residents’ “eyes and ears”. EFCs felt personally responsible for overseeing the care and advocating for their loved ones in LTCHs. When they were unable to fulfill their duties, they experienced negative emotional and behavioural consequences that overwhelmed their capacity to cope. One EFC described how the fear about her mother’s safety prevented her from sleeping:

With no extra staff and knowing how short staffed they are all the time. To kick us out, at a time when they would need us more, was unbelievably frightening. Every single night obsessing about [my mother] and it was impossible to sleep. It was constantly thinking [about] what’s going on […] it was absolutely frightening when we were kicked out and we weren’t in there to advocate. (EFC ‘2’, FG1)

Another EFC described how her mother had a medical history of choking on her food, and how she would visit at mealtimes to ensure her mother was safe while eating. The participant put in a request to maintain mealtime visitations to prevent choking but her request was denied and within three months her fears became a reality:

So then, in June, one morning at breakfast she choked, they had to perform CPR and suction and I was called, and I was allowed a one-hour compassionate visit […] then I was told my hour was over. I was back to window visits the next day, and the staff were crying […] [the staff] was just in tears, she said, ‘we almost lost her’ (EFC ‘1’, FG1)

This EFC became visibly upset when retelling the series of events that led to her mother’s “death scare”. The EFCs consistently became emotional during the FGs as they shared their experience of joy and horror when seeing their loved ones for the first time in months. Specifically, they saw significant physical and cognitive declines of their loved ones upon reunification that included a shift from walking independently to becoming wheelchair-bound, an inability to verbally communicate and noticeable weight losses. In some cases, EFCs reported their loved ones losing upwards of 30 pounds during the first three months of the pandemic, and described the appearance of their loved ones as “skin and bones”. As two participants reveal..

[My mother’s] speech deteriorated fairly rapidly, as well, because […] when coming in […] I would prompt her to talk to maintain the speech that she has. (EFC ‘3’, FG2)

I’ve even forgotten how much weight [my husband] lost, but he was just a skeleton and being so tall he look like a stick insect. He’d lost about 20 or 30 pounds because he wasn’t eating and when I went for a window visit once and look through the window all the other residents looked absolutely terrible, it was like looking through the window of bedlam. He was skeletal. (EFC ‘5’, FG5)

For some EFCs, rapid cognitive decline was the most noticeable form of deterioration. The cumulative impact of resident confinement and prolonged periods between visitations left residents without cognitive and social stimulation, which hitherto was provided primarily by their EFCs. One EFC, whose mother was in an LTCH due to early-onset Alzheimer’s, revealed the pain of seeing how much worse off her mother was once she was granted indoor access:

So, I got to see my mom again in September […] but she doesn’t know who I am anymore […] it’s hell for her, she hates life, she’s angry, she yells at the PSW […] I think she thinks [my sister and I] abandoned her and she is dying of a broken heart. (EFC ‘3’, FG3)

With EFCs unable to fulfill their typical caregiving duties, many were left with feelings of immense self-guilt and disappointment towards LTCHs for their lack of recognition of the vital care they provide.

### Theme 4: trauma from experiencing prolonged powerlessness and helplessness

EFCs were sensitive to the broader power imbalance between LTCHs and EFCs during the lockdown and were left feeling powerless and helpless. The LTC sector’s authority to suddenly implement policies that violated residents’ rights to access their EFCs, the paternalistic oversight of EFCs while inadequately monitoring staff who were commonly the source of COVID-19 outbreaks, inconsistent application of provincial guidelines between homes, and a fear of retaliation from LTCH staff were all contributors to EFCs feelings of helplessness. Underpinning their sense of powerlessness and helplessness was being invisible to governments and LTCHs during the early months of the pandemic, even being deemed “non-essential’ and classifying EFCs as general visitors during the first wave (Ontario) and well into the second wave (British Columbia). One participant expressed her helplessness and desperation to be allowed in to see her loved one:

Before [being an EFC and being locked out] I was so angry and helpless and hopeless. I would have done anything [to get in to see my husband] […] I would have bathed in sanitizer; I would have injected myself with bleach. (EFC ‘5’, FG5)

Additionally, EFCs described LTCHs as “playing God” which was a powerful metaphor depicting hierarchical differences felt by EFCs. Some participants who raised concerns to the LTCH staff perceived that they were chided as a “squeaky wheel” for bringing attention to issues related to neglect and improper care experienced by their loved ones. Many noted experiencing either reduced visitation access, revoked visitation status, or increasingly limited communication from the homes as a result. Accordingly, their fear of punitive treatments led to a sense of powerlessness and self-silencing:

You’re at the mercy of someone who holds all the power of when you can see your beloved family member next, and you want to complain, but you know if you complain, it might get worse, or you might have different rules than somebody else, or maybe grandma’s goanna suffer if you say something, so you just keep your mouth shut and wait when you’re allowed to visit next. (EFC ‘4’, FG3)

We finally got one Zoom call […] it didn’t happen [because] they took her for a shower during the weekly Zoom call but they forgot to tell me that […] I thought [my mother] was dead, so when I phoned the floor nurse she got very upset with me for being very angry and I said, ‘could you just go down the hall and make sure my mom is alive, because she didn’t show up for her call.’ They literally hung up on me instead of checking. (EFC ‘2’, FG4)

Within the context of the lockdown policies, EFCs had to completely rely on the LTCHs to keep their loved ones alive and well without any access to provide oversight. They felt powerless because they were unable to help or protect their loved ones. As one participant put it, the LTCHs struggled to provide basic care, and made a calculated decision to focus on “liability first and the humanity second […] the policies are set up for [the LTCH’s] liability. If [the LTCH] can keep [residents] physically alive then [the homes have] done what [they] need to do.” (EFC “3”, FG2)

When in-person visitations were initiated in LTCHs, EFCs were subjected to new and aggressive testing protocols levied by governments. Interestingly, these targeted testing policies were not required of other groups of healthcare professionals, including those working in both acute care and LTC. For EFCs granted outdoor and indoor access in Ontario, bi-weekly and weekly COVID-19 testing (dependent on the level of community transmission risk) was required. While EFCs were willing to get tested, it was a “draining” and painful, time-consuming process further muddied by the fact it was only provided by select locations which meant additional planning and long wait-times. Many participants reported completing more than 50 COVID-19 tests between June 2020 and February 2021. For those living in areas of higher community transmission (e.g., Toronto and Peel regions in Ontario), EFCs cited having to be tested twice weekly in order to get their results back in time for their weekly negative test attestation to the home’s management:

I’ve been going twice a week [to get my COVID test] […] [at] a family level I’m sort of the canary in the coal mine, so if I test positive, then we worry about the rest of the family, but as long as I’m okay, since I’m going, nobody else has to really worry. (EFC ‘Queen’, FG1)

I was [getting tested] once a week […] at first it was taking four days [to get the results back], and you know go figure, we are affiliated with the hospital you think we could have gotten a quicker turnaround on our results, but we didn’t. (EFC ‘2’, FG3)

EFCs were willing to do what was required of them by the LTCHs to see their relatives. In those first months of in-person visits, there was a strong security presence to enforce masking and physical distancing which troubled EFCs (Frketich, 2021). This underscored the vulnerability and “outsider” position of EFCs who were unable to show their loved ones their face even when physically distanced or even touch hands for a moment despite wearing gloves and using hand sanitizer before and after.

## Discussion

Our study examined the traumatic experiences of EFCs in Canada during COVID-19 whose access to their loved ones in LTCHs were restricted, and four themes were identified: trauma from prolonged separation from loved ones, trauma from uncompassionate interactions with the LTCH’s staff and administrators, trauma from the inability to provide care to loved ones, and trauma from experiencing prolonged powerlessness and helplessness. These themes are related but distinct sources of trauma that was consistent across all our FGs indicating that the effects of COVID-19 lockdown policies are likely to have caused long-lasting and irreparable damage for caregivers. Our findings are timely and relevant given that the pandemic is still ongoing in Canada with many homes entering and exiting lockdown COVID-19 outbreaks; therefore, these harmful policies may be reinstated and may be employed again in future waves or pandemics.

The lockdown restrictions exacerbated a confluence of long-standing issues in LTCHs—namely, high staff turnover (Chu et al., 2014), lower pay and devaluation of staff work (McGilton et al., 2020), and old buildings with antiquated infrastructures (Chu, Ronquillo et al., 2021) resulted in scared and neglected residents who were left “alone and cut off from those that they love and depend on” (Marrocco et al., 2021, p. 48). Remaining staff who continued working did so under incredibly dire and poor conditions (Webster, 2021) which likely contributed to oversights in communication reflected in our study. Recent research elucidated how the aforementioned context in LTC in addition to structural inequities impacted EFCs ability to access and use technologies during the lockdowns (Chu et al., 2022). EFCs felt powerless and hopeless in the situation without any allies in the LTCHs or the government.

This research has policy and practice implications. Firstly, there is a clear need for provincial and federal legislation barring the prevention of FCGs from family members in LTCHs. For example, Bill 203 attempted to accomplish this goal in Ontario, however it was not passed into law. Another policy implication, is that there is an ongoing conversation about the creation of national standards for LTCHs with two committees stuck to create draft standards. There is no obligation for provinces to follow any drafted national standards, and the current versions do not explicitly protect the FCG and resident relationships or the uninterrupted access to residents in LTC at the home level. These reports have yet to materialize into national standards so there still remains an opportunity to legislate caregiver protections.

Second, the negative emotional trauma and stress elicited by the pandemic lockdown policies highlight the need to build a collaborative LTC health system where informal caregivers (e.g., families) are empowered through being consistently involved in the health policy decision-making, updated on care practices/plans/guidelines, and supported socially and emotionally. One such practice model is one from the Veterans Affairs in the U.S. (Dang et al., 2020) where a proactive team approach was utilized to tailor outreach and communication to family members in order to identify, screen, support, educate, coordinate care, for high-risk residents and their caregivers. A vital part of a collaborative LTC system is ensuring adequate investments into digital resources, that will facillitate timely and effective communication at a home level (Chu et al., 2022; Chu, Ronquillo et al., 2021).

Third, empowering and mandating family councils across long-term care could strengthen the voice of families in decision-making. Family councils are self-led and self-determining groups of family and friends seeking to improve residents’ quality of life and improve LTC systems and procedures (Baumbucsch et al., 2022). Strengthening the capacity of family councils to engage in LTC decision making is an important area to address going forward. In July 2021, the province of Ontario increased funding to support the Ontario Association of Residents’ Councils (OARC) and Family Councils Ontario (FCO; Government of Ontario, 2021b). All provinces and territories should aim to mandate and sufficiently fund the creation and empowerment of family councils across Canada.

A practice implication is the application of a trauma-informed approach for all staff and administrators as they provide care for the residents and interact with EFCs who have both experienced collective trauma due to the COVID-19 policies in place. A trauma-informed approach is supported by the European Society for Trauma Stress Studies (ESTSS; Javakhishvili et al., 2020). The pandemic affected the whole of society in a multi-layered manner and disproportionately impacted vulnerable people, including individuals living in LTCHs and those caring for them. There are identified COVID-19 related stressors leading to psychological trauma which include: uncertainty in life circumstances; restricted social connection; imposed quarantines that oppose human rights such as freedom of movement; stigmatization, discrimination and fragmentation of communities; loss of loved ones; inability to conduct culturally appropriate mourning rituals; and finally, the threat of contracting or infecting others with COVID-19 (Javakhishvili et al., 2020). Given the range of stressors, there is a moral imperative to address the mental health and psychosocial wellbeing of the vulnerable groups, such as residents and EFCs. Trauma-informed care provides a framework that begins to create a system that fosters safety, trust through transparency, peer support, mutuality through collaboration and addressing power imbalances, empowerment through allowing people to have a voice and choice, and inclusivity (Substance Abuse and Mental Health Services Administration [SAMHSA], 2014). This initiative can be instigated by first training key staff members in trauma informed care (Javakhishvili et al., 2020) and forming peer-support services for staff and/or EFCs to discuss their concerns (SAMHSA, 2014). In Canada, this trauma informed approach is supported by the Ontario’s LTC Commissioner final report which recommended that LTCHs provide counselling assistance to staff and residents due to the fact “many continue to be traumatized as a result of this [lockdown] experience and will require ongoing counselling and support” (Marrocco et al., 2021, p. 19). The results of our qualitative research are corroborated by the high levels of stress-related symptomology reported by ECFs which indicate that the same consideration for supports should be extended to EFCs given the real and multifaceted nature of their trauma.

The othering of EFCs was fortified in policy that identified them as “non-essential visitors”, and within the LTCHs when security gaurds were hired to enforce visitation rules for EFCs (Frketich, 2021; Levy, 2021). LTCHs were positioned in power and EFCs were unable to have any say or control over their ability to visit. During COVID-19, our participants felt silenced, devalued and underappreciated for the vital role they play in LTCHs in caring for their loved ones. More importantly, they have felt abandoned and unrecognized by the broader healthcare system (Lilly et al., 2012), consistent with the caregiving literature that describes caregivers as “outsiders” and “invisible”. ECFs can experience vicarious traumatization (McCann & Pearlman, 1990), trauma caused by caring for others exposed traumas (e.g., the conditions in the LTC homes during these lockdown policies; Masiero et al., 2020). EFCs’ traumatic experiences have translated into negative physical and emotional symptoms (e.g., muscle tension, sleep loss, exhaustion, depression, panic attacks); as a consequence, of being unable to access their loved ones in LTCHs.

During the FGs, numerous participants thanked us for listening and for “giving them a voice”, and some participants shared that they are seeking professional counselling to cope with the trauma of being separated from their loved ones. This is concerning given that these perceptions reflect their vulnerability. An underlying cause of the trauma was not being heard or seen throughout the multiple waves of the pandemic. Our findings highlight a complex interrelationship between EFC’s and LTCHs, whereby EFCs were dependent on the LTCHs and that the status of an EFC was determined by the broader structural hierarchy of power. Additional macro-level changes to empower EFCs include a need to integrate public health and community-led social support groups for EFCs to ensure their voices, trauma, and concerns are being heard and respected. In doing so, the opportunity to share their experiences in a safe space helps validate their emotions and concerns (Abendroth et al., 2014) and allows them to feel a sense of broader connection to counter their feelings of isolation that was noted by some of our EFCs. As well as, create mechanisms and communication channels within our healthcare system to allow EFCs to report incidents to hold LTCHs accountable. Greater protections for staff should also be considered, but we recognize that such a balance will require broad community engagement to ensure a safe home and work environment.

### Strengths and limitations

A strength of the study is the timeliness of the data as the FGs took place during COVID-19, while visitation restrictions and guidelines were constantly being updated. Furthermore, to facilitate transparency of our methodology and add to the credibility of our study, we followed the COREQ checklist (Tong et al., 2007) in the reporting of this study (supplementary file). Additional strategies that added to the trustworthiness of the study were employed: researchers kept an audit trail of the field notes, research decisions and activities to ensure confirmability; multiple researchers (CC, AY, VS) analysed the same data independently and compared results to discuss and modify codes as part of dependability (Anney, 2014). We are confident that our findings are transferable to other LTC home settings across Ontario and BC, and have provided thick descriptions of the details and context (Anney, 2014) throughout the results section and in the coding tree. Additionally, one of the PIs has had prolonged engagement in the field (Anney, 2014) and has been deeply entrenched in working alongside and advocating for FCGs for the entirety of the pandemic in Ontario and BC providing a deep understanding of the topic. However, each province managed the pandemic in LTCHs differently so the results may not be transferable to EFCs living in the other provinces.

Due to provincial guideline at the time that restricted all in-person gatherings between households or “bubbles”, the researchers had no option other than to conduct virtual focus groups. Reflecting on utilizing virtual focus groups to follow these provincial guidelines at the height of the COVID-19 pandemic, we had concerns about privacy and poor internet connectivity. The researchers made sure to send out calendar invites, send out reminder emails, instructed participants to relocate to a private space in their home for the focus group. Fortunately, we did not experience any issues with connectivity, privacy, or no-shows to the online FGs.

This study is not without limitations. One limitation is that despite PIs’ efforts to recruit male participants, only one participant was male. This is unsurprising as it reflects the reality that informal caregiving for older family members has long been a role primarily and disproportionately held by women (National Partnership for Women & Families, 2018). Also, we only included family caregivers who spoke English, and the majority were Caucasian. This is a limitation as this study was not able to capture the experiences of non-English speaking caregivers or other types of caregivers that were designed by the residents (e.g., friends). Future research that is inclusive of other languages and cultures is warranted as their experiences with the healthcare system may differ from individuals who are Caucasian and speak English. Another limitation is that we did not inquire about whether the honorarium influenced recruitment. However, the electronic gift card amount was approved by both universities’ research ethic boards and was deemed standard renumeration that would not create an undue pressure for adults to participate. In discussions between the investigators, it was clear that many of the participants appeared to forget that they were going to receive an honorarium and were content to share their experiences and that they would have done so without any form of honorarium. Finally, the researchers stated to participants prior to the start of the FGs that they may experience emotional harm from reliving their difficult experiences and hearing others recount similar stories. The researchers made it clear that participants could take a break at any time and we had a list of trauma support and distress related resources that we offered to all participants before and after the focus groups. Although some participants became visibly upset during parts of the discussion, no participants wanted to take a break or requested any support services. During the FGs, the participants mentioned that connecting with other EFCs who had also experienced being separated from their relative in LTC was gratifying and provided a level of validation for their own feelings. Given these benefits, it may be helpful to arrange post-focus group support sessions for interested participants in a future study. The findings of our study offer valuable insights to better understand the harms caused by these policies as well as how to improve pandemic health policies in LTCH moving forward. Future research should include dyadic methodologies that combine the residents’ and caregivers’ perspectives, the experiences of ECs who are not family, as well as expanding our understanding of the long-term psychological and behavioural consequences of this trauma in residents and caregivers.

## Conclusion

Our study on the traumatic experiences of EFCs with loved ones living in LTCHs during COVID-19 illustrates the tremendous impact of the lockdown policies on EFCs. The results of this research highlight the detrimental effects of the lockdown policies on residents in LTCHs and EFCs. A significant amount of work is needed to heal and mitigate the long-term damage between the family’s relationship with their loved ones and the family’s mistrust of the LTC system. Understanding the stories of EFCs during the COVID-19 pandemic can inform future approaches to holistically evaluate policies that seek to restrict access and support to those living LTCHs and other congregate care facilities. More thoughtful regulations and legislation aimed at enforcing and strengthening existing rights of EFCs and LTCH residents to maintain access and maintain their relationships must occur for this setting.

## Supplementary Material

Supplemental Material

## References

[cit0001] Abbasi, J. (2020). Social isolation—The other COVID-19 threat in nursing homes. *JAMA*, 324(7), 619–17. 10.1001/jama.2020.13484 32692848

[cit0002] Abendroth, M., Greenblum, C. A., & Gray, J. A. (2014). The value of peer-led support groups among caregivers of persons with Parkinson’s disease. *Holistic Nursing Practice*, 28(1), 48–54. 10.1097/HNP.0000000000000004 24304631

[cit0003] Ae-Ngibise, K. A., Doku, V. C. K., Asante, K. P., & Owusu-Agyei, S. (2015). The experience of caregivers of people living with serious mental disorders: A study from rural Ghana. *Global Health Action*, 8(1), 1. 10.3402/GHA.V8.26957 PMC4429259 25967587

[cit0004] Anney, V. N. (2014). Ensuring the quality of the findings of qualitative research: Looking at trustworthiness criteria. *Journal of emerging trends in educational research and policy studies*, 5(2), 272–281.

[cit0005] Baumbusch, J., & Phinney, A. (2014). Invisible hands: The role of highly involved families in long-term residential care. *Journal of Family Nursing*, 20(1), 73–97. 10.1177/1074840713507777 24122579

[cit0006] Baumbusch, J., Sloan Yip, I., Koehn, S., Reid, R. C., & Gandhi, P. (2022). A Survey of the Characteristics and Administrator Perceptions of Family Councils in a Western Canadian Province. *Journal of Applied Gerontology*, 41(2), 363–370. 10.1177/0733464820961257 32996401

[cit0007] Braun, V., & Clarke, V. (2006). Using thematic analysis in psychology. *Qualitative Research in Psychology*, 3(2), 77–101. 10.1191/1478088706qp063oa

[cit0008] Canadian Institute for Health Information. (2021). The impact of COVID-19 on long-term care in Canada: Focus on the first 6 months. https://www.cihi.ca/sites/default/files/document/impact-covid-19-long-term-care-canada-first-6-months-report-en.pdf

[cit0009] Chu, C. H., Wodchis, W. P., & McGilton, K. S. (2014). Turnover of regulated nurses in long-term care facilities. *Journal of Nursing Management*, 22(5), 553–562. 10.1111/jonm.12031 25041798

[cit0010] Chu, C. H., Donato-Woodger, S., & Dainton, C. J. (2020). Competing crises: COVID-19 countermeasures and social isolation among older adults in long-term care. *Journal of advanced nursing*, 76(10), 2456–2459. 10.1111/jan.14467 32643787 PMC7361866

[cit0011] Chu, C. H., Donato‐Woodger, S., & Dainton, C. J. (2020). Competing crises: COVID‐19 countermeasures and social isolation among older adults in long‐term care. *Journal of Advanced Nursing*, 76(10), 2456–2459. 10.1111/jan.14467 32643787 PMC7361866

[cit0012] Chu, C. H., Wang, J., Fukui, C., Staudacher, S., Wachholz, A., & Wu, B. (2021). The impact of COVID-19 on social isolation in long-term care homes: Perspectives of policies and strategies from six countries. *Journal of Aging & Social Policy* 33(4–5) , 1–15. 10.1080/08959420.2021.1924346 33969815

[cit0013] Chu, C. H., Wang, J., Fukui, C., Staudacher, S., Wachholz, A., & Wu, B. (2021). The impact of COVID-19 on social isolation in long-term care homes: Perspectives of policies and strategies from six countries. *Journal of Aging & Social Policy*, 5(33), 1–15. 10.1080/08959420.2021.1924346 33969815

[cit0014] Chu, C. H., Ronquillo, C., Khan, S., Hung, L., & Boscart, V. (2021). Technology recommendations to support person-centered care in long-term care homes during the COVID-19 pandemic and beyond. *Journal of Aging & Social Policy*, 33(4–5), 539–554. 10.1080/08959420.2021.1927620 34278980

[cit0015] Chu, C. H., Yee, A., & Stamatopoulos, V. (2022). Poor and Lost Connections: Essential Family Caregivers’ Experiences Using Technology with Family Living in Long-Term Care Homes during COVID-19. *Journal of Applied Gerontology*, 41(6), 1547–1556. 10.1177/07334648221081850 35416076 PMC9014337

[cit0016] CTV Ottawa. (2020). Caregivers seek to be essential service. *CTV News*. https://ottawa.ctvnews.ca/video?clipId=1992926

[cit0017] Dang, S., Penney, L. S., Trivedi, R., Noel, P. H., Pugh, M. J., Finley, E., Pugh, J. A., Van Houtven, C. H., & Leykum, L. (2020). Caring for caregivers during COVID‐19. *Journal of the American Geriatrics Society*, 68(10), 2197–2201. 10.1111/JGS.16726 PMC7361597 32638348

[cit0018] Diamantis, S., Noel, C., Tarteret, P., Vignier, N., & Gallien, S. (2020). Severe acute respiratory syndrome coronavirus 2 (SARS-CoV-2)-related deaths in French long-term care facilities: The “confinement disease” is probably more deleterious than the coronavirus disease-2019 (COVID-19) itself. *Journal of the American Medical Directors Association*, 21(7), 989–990. 10.1016/j.jamda.2020.04.023 32507530 PMC7196427

[cit0019] Drury, J. (2020, April 20). family caregivers as essential partners in care: More than just a visitor. Canadian Foundation for Healthcare Improvement. https://www.cfhi-fcass.ca/about/news-and-stories/news-detail/2020/04/20/family-caregivers-as-essential-partners-in-care-more-than-just-a-visitor

[cit0020] Estabrooks, C. A., & Keefe, J. (2020, May 19). COVID-19 crisis in nursing homes is a gender crisis. Policy Options. https://policyoptions.irpp.org/magazines/may-2020/covid-19-crisis-in-nursing-homes-is-a-gender-crisis/

[cit0021] Faghanipour, S., Monteverde, S., & Peter, E. (2020). COVID-19-related deaths in long-term care: The moral failure to care and prepare. *Nursing Ethics*, 27(5), 1171–1173. 10.1177/0969733020939667 32703121

[cit0022] Figley, C. R. (1995). Compassion fatigue: Toward a new understanding of the costs of caring. In B. H. Stamm (Ed.), *Secondary traumatic stress: Self care issues for clinicians, researchers, and educators* (pp. 3–28). Sidran Press.

[cit0023] Frketich, J. (2021, January 28). Security guards at doors of LTC homes ‘chilling,’ says NDP leader. *The Hamilton Spectator*. https://www.thespec.com/news/hamilton-region/2021/01/28/security-guards-at-doors-of-ltc-homes-chilling-says-ndp-leader.html

[cit0024] Garity, J. (2006). Caring for a family member with Alzheimer’s disease: Coping with caregiver burden post-nursing home placement. *Journal of Gerontological Nursing*, 32(6), 39–48. 10.3928/00989134-20060601-07 16773862

[cit0025] Gaugler, J. E., Leitsch, S. A., Zarit, S. H., & Pearlin, L. I. (2000). Caregiver involvement following institutionalization: Effects of preplacement stress. *Research on Aging*, 22(4), 337–359. 10.1177/0164027500224002

[cit0026] Gaugler, J. E. (2005). Family involvement in residential long-term care: A synthesis and critical review. *Aging & Mental Health*, 9(2), 105–118. 10.1080/13607860412331310245 15804627 PMC2247412

[cit0027] Gladstone, J. W., Dupuis, S. L., & Wexler, E. (2006). Changes in family involvement following a relative’s move to a long-term care facility. *Canadian Journal on Aging*, 25(1), 93–106. 10.1353/CJA.2006.0022 16770751

[cit0028] Glaser, B. G., & Strauss, A. L. (1967). *Discovery of grounded theory: Strategies for qualitative research*. Aldine Transaction: A Division of Transaction Publishers.

[cit0029] Goldkuhl, G. (2012). Pragmatism vs interpretivism in qualitative information systems research. *European Journal of Information Systems*, 21(2), 135–146. 10.1057/ejis.2011.54

[cit0030] Government of British Columbia. (2020, March 16). Joint statement on B.C.’s COVID-19 response and latest updates. *BC Government News*. https://news.gov.bc.ca/releases/2020HLTH0086-000499

[cit0031] Government of British Columbia. (2021, March 25). COVID-19: Visitor restrictions in long-term care & seniors’ assisted living. *British Columbia (BC) Government News*. https://news.gov.bc.ca/files/3.25.2021_Easing_Restrictions_LTC.pdf

[cit0032] Government of Ontario. (2021a). COVID-19 guidance document for long-term care homes in Ontarioo. https://www.ontario.ca/page/covid-19-guidance-document-long-term-care-homes-ontario#section-11

[cit0033] Government of Ontario. (2021b). Ontario boosting funding to long-term care resident and family councils. https://news.ontario.ca/en/release/1000572/ontario-boosting-funding-to-long-term-care-resident-and-family-councils

[cit0034] Gretzky, L. (2020). Bill 203, more than a visitor act (Caregiving in congregate care settings). Legislative Assembly of Ontario. https://www.ola.org/en/legislative-business/bills/parliament-42/session-1/bill-203

[cit0035] Griffin, G. (2020). Defining trauma and a trauma-informed COVID-19 response. *Psychological Trauma: Theory, Research, Practice, and Policy*, 12(S1), S280. 10.1037/TRA0000828 32551754

[cit0036] Hado, E., & Feinberg, L. F. (2020). Amid the COVID-19 pandemic, meaningful communication between family caregivers and residents of long-term care facilities is imperative. *Journal of Aging & Social Policy*, 32(4–5), 410–415. 10.1080/08959420.2020.1765684 32441209

[cit0037] Harris, S. (2020, August 13). Ontario families fight for more long-term care visits before 2nd wave hits | HuffPost Canada News. *HuffPost*. https://www.huffingtonpost.ca/entry/ontario-covid-long-term-care-family-visits_ca_5f353edac5b6960c06718c4c

[cit0038] Hirschberger, G. (2018). Collective trauma and the social construction of meaning. *Frontiers in Psychology*, 9, 1–14. 10.3389/fpsyg.2018.01441 30147669 PMC6095989

[cit0039] Holt-Lunstad, J., Smith, T. B., Baker, M., Harris, T., & Stephenson, D. (2015). Loneliness and social isolation as risk factors for mortality: A meta-analytic review. *Perspectives on Psychological Science*, 10(2), 227–237. 10.1177/1745691614568352 25910392

[cit0040] Howlett, K. (2021, May 9). Patients died from neglect, not COVID-19, in Ontario LTC homes, military report finds: ‘All they needed was water and a wipe down’. *The Globe and Mail*. https://www.theglobeandmail.com/canada/article-canadian-military-report-documents-deplorable-conditions-at-two/

[cit0041] Hübl, T., & Avritt, J. J. 2020. *Healing collective trauma: A process for integrating our intergenerational and cultural wounds*. Sounds True.

[cit0042] Javakhishvili, J. D., Ardino, V., Bragesjö, M., Kazlauskas, E., Olff, M., & Schäfer, I. (2020). Trauma-informed responses in addressing public mental health consequences of the COVID-19 pandemic: Position paper of the European Society for Traumatic Stress Studies (ESTSS). *European Journal of Psychotraumatology*, 11(1), 1. 10.1080/20008198.2020.1780782 PMC7473312 33029320

[cit0043] Kirkup, K. (2020, May 26). Canadian military releases ‘deeply disturbing’ report on Ontario long-term care facilities. *The Globe and Mail*. https://www.theglobeandmail.com/politics/article-trudeau-ford-discuss-deeply-disturbing-canadian-forces-report-on/

[cit0044] Krueger, R. A., & Casey, M. A. (2014). *Focus groups: A practical guide for applied research* (p. 280). Sage publications. .

[cit0045] Levy, S.-A. (2021, February 4). LEVY: Advocates livid over $42M for LTC home security guards. *Toronto Sun*. https://torontosun.com/news/local-news/levy-advocates-livid-over-42m-for-ltc-home-security-guards

[cit0046] Lilly, M. B., Robinson, C. A., Holtzman, S., & Bottorff, J. L. (2012). Can we move beyond burden and burnout to support the health and wellness of family caregivers to persons with dementia? Evidence from British Columbia, Canada. *Health & Social Care in the Community*, 20(1), 103–112. 10.1111/j.1365-2524.2011.01025.x 21851447

[cit0047] Liu, M., Maxwell, C. J., Armstrong, P., Schwandt, M., Moser, A., McGregor, M. J., Bronskill, S. E., & Dhalla, I. A. (2020). COVID-19 in long-term care homes in Ontario and *British Columbia*. *Canadian Medical Association Journal*, 192(47), E1540–E1546. 10.1503/cmaj.201860 32998943 PMC7721263

[cit0048] Lund, E. M., Forber-Pratt, A. J., Wilson, C., & Mona, L. R. (2020). The COVID-19 pandemic, stress, and trauma in the disability community: A call to action. *Rehabilitation Psychology*, 65(4), 322. 10.1037/REP0000368 33119381

[cit0049] Mahoney, J. (2020, June 3). What happened when families were blocked from Canada’s long-term care homes. *The Globe and Mail*. https://www.theglobeandmail.com/canada/article-what-happened-when-families-were-blocked-from-long-term-care-homess/

[cit0050] Marrocco, F. N., Coke, A., & Kitts, J. (2021). Ontario’s long-term care COVID-19 commission: Final report. http://www.ltccommission-commissionsld.ca/report/pdf/Ontarios_Long-Term_Care_COVID-19_Commission_Final_Report.pdf

[cit0051] Masiero, M., Mazzocco, K., Harnois, C., Cropley, M., & Pravettoni, G. (2020). From individual to social trauma: Sources of everyday trauma in Italy, the US and UK during the COVID-19 pandemic. *Journal of Trauma and Dissociation*, 21(5), 1–7. 10.1080/15299732.2020.1787296 32654633

[cit0052] Mauro, E. (2020, July 11). It’s time to let family caregivers back into Ontario nursing homes, medical officer says. *CBC News*. https://www.cbc.ca/news/canada/ontario-nursing-homes-covid-19-1.5644621

[cit0053] McCann, I. L., & Pearlman, L. A. (1990). Vicarious traumatization: A framework for understanding the psychological effects of working with victims. *Journal of Traumatic Stress*, 3(1), 131–149. 10.1007/BF00975140

[cit0054] McGilton, K. S., Escrig-Pinol, A., Gordon, A., Chu, C. H., Zúñiga, F., Sanchez, M. G., Boscart, V., Meyer, J., Corazzini, K. N., Jacinto, A. F., Spilsbury, K., Backman, A., Scales, K., Fagertun, A., Wu, B., Edvardsson, D., Lepore, M. J., Leung, A. Y. M., Siegel, E. O., … Bowers, B. (2020). Uncovering the devaluation of nursing home staff during COVID-19: Are we fuelling the next health care crisis? *Journal of the American Medical Directors Association*, 21(7), 962–965. 10.1016/J.JAMDA.2020.06.010 32674829 PMC7287421

[cit0055] Ministry of Long-Term Care. (2020, September 2). Resuming visits in long-term care homes. https://www.health.gov.on.ca/en/pro/programs/ltc/docs/covid-19/mltc_visitor_policy_20200909_en.pdf

[cit0056] Morciano, M., Stokes, J., Kontopantelis, E., Hall, I., & Turner, A. J. (2021). Excess mortality for care home residents during the first 23 weeks of the COVID-19 pandemic in England: A national cohort study. *BMC Medicine*, 19(1), 71. 10.1186/s12916-021-01945-2 33663498 PMC7932761

[cit0057] National Academies of Sciences, Engineering, and Medicine. (2016). Families caring for an aging America. National Academies Press. 10.17226/23606 27905704

[cit0058] National Academies of Sciences, Engineering, and Medicine. (2020). *Social isolation and loneliness in older adults: Opportunities for the health care system*. National Academies Press. 10.17226/25663 32510896

[cit0059] National Institute on Ageing. (2020). The NIA’s ‘iron ring’ guidance for protecting older Canadians in long-term care and congregate living settings.

[cit0060] National Partnership for Women & Families. (2018, November). The female face of family caregiving. https://www.nationalpartnership.org/our-work/resources/economic-justice/female-face-family-caregiving.pdf

[cit0061] The New York Times. (2020, December 29). Nursing home patients are dying of loneliness. https://www.nytimes.com/2020/12/29/opinion/coronavirus-nursing-homes.html

[cit0062] Novotney, A. (2019). The risks of social isolation. *Monitor on Psychology*, 50, 5. https://www.apa.org/monitor/2019/05/ce-corner-isolation

[cit0063] O’Connor, A., Jackson, L., Goldsmith, L., & Skirton, H. (2014). Can I get a retweet please? Health research recruitment and the Twittersphere. *Journal of Advanced Nursing*, 70(3), 599–609. 10.1111/jan.12222 23909740

[cit0064] Patient Ombudsman. (2020). Special report: Honouring the voices and experiences of long-term care home residents, caregivers and staff during the first wave of COVID-19. https://www.patientombudsman.ca/Portals/0/documents/covid-19-report-en.pdf

[cit0065] Port, C. L., Zimmerman, S., Williams, C. S., Dobbs, D., Preisser, J. S., & Williams, S. W. (2005). families filling the gap: Comparing family involvement for assisted living and nursing home residents with dementia. *The Gerontologist*, 45(Suppl. 1), 87–95. 10.1093/GERONT/45.SUPPL_1.87 16230755

[cit0066] Powell, C., Blighe, A., Froggatt, K., McCormack, B., Woodward-Carlton, B., Young, J., Robinson, L., & Downs, M. (2018). Family involvement in timely detection of changes in health of nursing homes residents: A qualitative exploratory study. *Journal of Clinical Nursing*, 27(1–2), 317–327. 10.1111/jocn.13906 28557103 PMC5767757

[cit0067] Reinhard, S. C., Feinberg, L. F., Houser, A., Choula, R., & Evans, M. (2019). Valuing the invaluable 2019 update: Charting a path forward. *AARP Public Policy Institute* 146 1–32 . 10.26419/PPI.00082.001

[cit0068] Roumeliotis, I., & Mancini, M. (2020, October 18). Restrictions on care home visits traumatize families, caregivers. *CBC News*. https://www.cbc.ca/news/health/pandemic-covid-caregivers-trauma-anxiety-1.5765027

[cit0069] Sagoe, D. (2012). Precincts and prospects in the use of focus groups in social and behavioral science research. *The Qualitative Report*, 17(15), 1–16. 10.46743/2160-3715/2012.1784

[cit0070] Schlaudecker, J. D. (2020). Essential family caregivers in long-term care during the COVID-19 pandemic. *Journal of the American Medical Directors Association*, 21(7), 983. 10.1016/j.jamda.2020.05.027 PMC7241354 32536552

[cit0071] Silver, R. C. (2020). Surviving the trauma of COVID-19. *Science*, 369(6499), 11. 10.1126/science.abd5396 32631871

[cit0072] Simard, J., & Volicer, L. (2020). Loneliness and isolation in long-term care and the COVID-19 pandemic. *Journal of the American Medical Directors Association*, 21(7), 966–967. 10.1016/j.jamda.2020.05.006 32505516 PMC7205644

[cit0073] Stall, N. M., Campbell, A., Reddy, M., & Rochon, P. A. (2019). Words matter: The language of family caregiving. *Journal of the American Geriatrics Society*, 67(10), 2008–2010. 10.1111/jgs.15988 31120551

[cit0074] Stall, N. M., Johnstone, J., McGeer, A. J., Dhuper, M., Dunning, J., & Sinha, S. K. (2020). Finding the right balance: an evidence-informed guidance document to support the re-opening of Canadian nursing homes to family caregivers and visitors during the coronavirus disease 2019 pandemic. *Journal of the American Medical Directors Association*, 21(10), 1365–1370.e7. 10.1016/j.jamda.2020.07.038 32981662 PMC7396877

[cit0075] Stanley, B. L., Zanin, A. C., Avalos, B. L., Tracy, S. J., & Town, S. (2021). Collective emotion during collective trauma: A metaphor analysis of the COVID-19 pandemic. *Qualitative Health Research*, 31(10), 1890–1903. 10.1177/10497323211011589 33980096

[cit0076] Statistics Canada. (2018). Care counts: Caregivers in Canada, 2018. https://www150.statcan.gc.ca/n1/pub/11-627-m/11-627-m2020001-eng.pdf

[cit0077] Substance Abuse and Mental Health Services Administration. (2014). SAMHSA’s concept of trauma and guidance for a trauma-informed approach. https://ncsacw.samhsa.gov/userfiles/files/SAMHSA_Trauma.pdf

[cit0078] Taggart, D., Rouf, K., Hisham, I. B. I., Duckworth, L., & Sweeney, A. (2021). Trauma, mental health and the COVID-19 crisis: Are we really all in it together? *Journal of Mental Health*, 30(4), 401–404. 10.1080/09638237.2021.1875415 33522346

[cit0079] Thomas, D. R. (2006). A general inductive approach for analysing qualitative evaluation data. *American Journal of Evaluation*, 27(2), 237–246. 10.1177/1098214005283748

[cit0080] Tong, A., Sainsbury, P., & Craig, J. (2007). Consolidated criteria for reporting qualitative research (COREQ): A 32-item checklist for interviews and focus groups. *International Journal for Quality in Health Care*, 19(6), 349–357. 10.1093/intqhc/mzm042 17872937

[cit0081] Van den Hoonaard, D. K., & Van den Scott, L. J. K. (2015). *Qualitative research in action: A Canadian primer*. Oxford University Press.

[cit0082] Webster, P. (2021). COVID-19 highlights Canada’s care home crisis. *The Lancet*, 397(10270), 183. 10.1016/S0140-6736(21)00083-0 PMC7833259 33453769

[cit0083] Williams, D. (2020). Chief medical officer of health memo – COVID-19 updates: Visitors. Government of Ontario. https://www.health.gov.on.ca/en/pro/programs/publichealth/coronavirus/docs/memos/CMOH_Memo_Visitors_COVID-19_March_13_2020.pdf

[cit0084] Wu, B. (2020). Social isolation and loneliness among older adults in the context of COVID-19: A global challenge. *Global Health Research and Policy*, 5(27) 1–3 . 10.1186/s41256-020-00154-3 32514427 PMC7272234

[cit0085] Zimmerman, S., Cohen, L. W., Reed, D., Gwyther, L. P., Washington, T., Cagle, J. G., Beeber, A. S., & Sloane, P. D. (2013). Comparing families and staff in nursing homes and assisted living: Implications for social work practice. *Journal of Gerontological Social Work*, 56(6), 535–553. 10.1080/01634372.2013.811145 23869592 PMC3772131

