# Supplementary material for: “It’s the worst thing I’ve ever been put through in my life”: the trauma experienced by essential family caregivers of loved ones in long-term care during the COVID-19 pandemic in Canada
Source: Int J Qual Stud Health Well-being. 2022 May 30;17(1):2075532. doi: 10.1080/17482631.2022.2075532 (PMC9176373; doi:10.1080/17482631.2022.2075532)
Supplement: Supplemental Material [file ZQHW_A_2075532_SM6925.docx]

**Table S1: Consolidated criteria for Reporting Qualitative research (COREQ) Checklist**

| **No. Item** | **Guide questions/description** | **Answers** | **page** |
| --- | --- | --- | --- |
| **Domain 1: Research team and reﬂexivity** |  |  |  |
| *Personal Characteristics* |  |  |  |
| 1. Interviewer/facilitator | Which author/s conducted the interview or focus group? | CC; VS | 11 |
| 2. Credentials | What were the researcher’s credentials? E.g. PhD, MD | CC is an RN, GNC(c), PhD; VS is a PhD | 11 |
| 3. Occupation | What was their occupation at the time of the study? | Both PIs are professors at Ontario universities. | 11 |
| 4. Gender | Was the researcher male or female? | Both PIs are female. | 11 |
| 5. Experience and training | What experience or training did the researcher have? | Both PIs are well versed in qualitative research and have published papers using qualitative methods. | 11 |
| *Relationship with participants* |  |  |  |
| 6. Relationship established | Was a relationship established prior to study commencement? | No. | 12 |
| 7. Participant knowledge of the interviewer | What did the participants know about the researcher? e.g. personal goals, reasons for doing the research | VS is a prominent advocate for and researcher of LTC families; CC is a researcher focused on improving the care of older adults in LTC. Both PIs have in-depth knowledge of LTCHs (e.g. CC conducted her PhD in LTC homes) | 31 |
| 8. Interviewer characteristics | What characteristics were reported about the interviewer/facilitator? e.g. Bias, assumptions, reasons and interests in the research topic | The interviewers both have PhD and are currently female professors at Ontario-based universities. | 11 |
| **Domain 2: study design** |  |  |  |
| *Theoretical framework* |  |  |  |
| 9. Methodological orientation and Theory | What methodological orientation was stated to underpin the study? e.g. grounded theory, discourse analysis, ethnography, phenomenology, content analysis | Interpretivist lens, whereby the FGs allowed participants to explain their lived experiences in their own words. Inductive thematic analysis informed the line-by-line coding of the data. | 12-13 |
| *Participant selection* |  |  |  |
| 10. Sampling | How were participants selected? e.g. purposive, convenience, consecutive, snowball | Purposive sampling was used, with the intent to recruit an equal number of male and female caregivers, but despite our efforts majority of the sample was female. | 9 |
| 11. Method of approach | How were participants approached? e.g. face-to-face, telephone, mail, email | Twitter, a social media platform, was used to recruit participants. Interested participants emailed the PIs and communication occurred primarily through email. | 9 |
| 12. Sample size | How many participants were in the study? | 30 | 10 |
| 13. Non-participation | How many people refused to participate or dropped out? Reasons? | 0 | 12 |
| *Setting* |  |  |  |
| 14. Setting of data collection | Where was the data collected? e.g. home, clinic, workplace | Virtually, over Zoom, a videoconferencing platform. | 10-11 |
| 15. Presence of non-participants | Was anyone else present besides the participants and researchers? | No. | 11-12 |
| 16. Description of sample | What are the important characteristics of the sample? e.g. demographic data, date | All were family caregivers of a loved one living in LTCH and during COVID-19 were unable to visit for extended periods of time. These family members self-identified as “essential caregivers”, provided emotional support and companionship for their loved ones, and can speak English. Characteristics of the EFCs and the LTCH facility their loved ones are staying in are provided in a table. | 9 and table 1 |
| *Data collection* |  |  |  |
| 17. Interview guide | Were questions, prompts, guides provided by the authors? Was it pilot tested? | Yes, a pilot-tested, semi-structured guide was used. | 12 |
| 18. Repeat interviews | Were repeat interviews carried out? If yes, how many? | None. | 12 |
| 19. Audio/visual recording | Did the researcher use audio or visual recording to collect the data? | Video and audio recording. | 12 |
| 20. Field notes | Were ﬁeld notes made during and/or after the interview or focus group? | Yes, field notes were made, and these were checked between each PIs after each FG. The notes also served to inform the initial generation of the codes. | 12 |
| 21. Duration | What was the duration of the interviews or focus group? | 90 minutes per FGs. | 8 |
| 22. Data saturation | Was data saturation discussed? | Yes, data saturation was reached after seven FGs. | 12 |
| 23. Transcripts returned | Were transcripts returned to participants for comment and/or correction? | No. | N/A |
| **Domain 3: analysis and ﬁndings** |  |  |  |
| *Data analysis* |  |  |  |
| 24. Number of data coders | How many data coders coded the data? | Three coders (CC, AY, VS) contributed to the coding, coding dictionary, and generation of the themes. | 13 |
| 25. Description of the coding tree | Did authors provide a description of the coding tree? | Yes, included as a figure. | Figure 1 |
| 26. Derivation of themes | Were themes identiﬁed in advance or derived from the data? | Inductive, as the themes were derived from the data. | 13 |
| 27. Software | What software, if applicable, was used to manage the data? | NVivo 12 software. | 13 |
| 28. Participant checking | Did participants provide feedback on the ﬁndings? | No. | N/A |
| *Reporting* |  |  |  |
| 29. Quotations presented | Were participant quotations presented to illustrate the themes/ﬁndings? Was each quotation identiﬁed? e.g. participant number | Yes. | 15-26 |
| 30. Data and ﬁndings consistent | Was there consistency between the data presented and the ﬁndings? | Yes. | 15-26 |
| 31. Clarity of major themes | Were major themes clearly presented in the ﬁndings? | Yes. | 15-26 |
| 32. Clarity of minor themes | Is there a description of diverse cases or discussion of minor themes? | Yes. | 15-26 |

Developed from: Tong A, Sainsbury P, Craig J. Consolidated criteria for reporting qualitative research (COREQ): a 32-item checklist for interviews and focus groups. International Journal for Quality in Health Care. 2007; 19, 349 – 357.
